# Non invasive ventilation as an additional tool for exercise training

**DOI:** 10.1186/s40248-015-0008-1

**Published:** 2015-04-09

**Authors:** Nicolino Ambrosino, Paolo Cigni

**Affiliations:** Pulmonary Rehabilitation and Weaning Center, Auxilium Vitae, Borgo S.Lazzaro 5, Volterra, PI Italy

**Keywords:** Chronic heart failure, COPD, Dyspnoea, Pulmonary rehabilitation

## Abstract

Recently, there has been increasing interest in the use of non invasive ventilation (NIV) to increase exercise capacity. In individuals with COPD, NIV during exercise reduces dyspnoea and increases exercise tolerance.

Different modalities of mechanical ventilation have been used non-invasively as a tool to increase exercise tolerance in COPD, heart failure and lung and thoracic restrictive diseases. Inspiratory support provides symptomatic benefit by unloading the ventilatory muscles, whereas Continuous Positive Airway Pressure (CPAP) counterbalances the intrinsic positive end-expiratory pressure in COPD patients.

Severe stable COPD patients undergoing home nocturnal NIV and daytime exercise training showed some benefits. Furthermore, it has been reported that in chronic hypercapnic COPD under long-term ventilatory support, NIV can also be administered during walking.

Despite these results, the role of NIV as a routine component of pulmonary rehabilitation is still to be defined.

## Background

During exercise in people with chronic obstructive pulmonary disease (COPD), expiratory flow limitation and increased respiratory frequency may reduce the expiratory time, resulting in increase in end-expiratory lung volume, a condition also known as dynamic hyperinflation (DH). In such condition, tidal breathing takes place at lung volumes closer to total lung capacity [1]. An unavoidable physiological consequence of DH is an increase in the intrinsic positive end-expiratory pressure (PEEPi) and in the elastic work of breathing (WOB): the final result is severe dyspnoea during effort reducing the exercise tolerance [2,3].

Pulmonary rehabilitation is a recognised core component of the comprehensive management of COPD patients, mainly based on exercise training [4]. In patients with chronic heart failure (CHF), exercise training has beneficial direct and reflex sympatho-inhibitory effects and favorable effects on normalisation of neurohumoral excitation. These physiological benefits apply to all COPD and CHF patients independently of the degree of disease severity and are associated with improved exercise tolerance, functional capacity, and quality of life [5].

Recently, there has been an increasing interest in the use of non invasive ventilation (NIV) to improve exercise capacity [6-8], based on the evidence that assisted ventilation, by unloading respiratory muscles, might allow patients to train at higher levels of exercise intensity. This review describes the present state of the art on the use of NIV as a tool to increase the benefits of pulmonary rehabilitation.

## Rationale

In a study on healthy trained cyclists it was shown that the WOB required to sustain high-intensity exercise significantly inversely correlated with leg blood flow [9,10]. In other words, during leg exercise, in conditions like COPD where WOB increases, there may be a switch of blood flow from limb to respiratory muscles, thus inducing earlier limb muscle fatigue, a well known cause of exercise interruption. This competition may be minimised by adding assisted ventilation during exercise. Confirming this hypothesis, in other studies assisted ventilation prevented exercise-induced diaphragmatic fatigue [11,12]. Because PEEPi and less effective inspiratory muscles in COPD contribute to dyspnoea, assisted ventilation should provide a symptomatic benefit by unloading and assisting the overburdened inspiratory muscles and reducing WOB. Unloading of the inspiratory muscles in turn may reduce their blood requirement allowing a switch of blood flow from respiratory to limb muscles.

Another aspect may be important. Systemic inflammation has been related to the development of comorbidities in COPD. The level of systemic inflammatory mediators is increased in response to exercise in these patients [13]. Non invasive ventilation may prevent the interleukin-6 response to exercise in muscle-wasted COPD patients [14]. Furthermore, sympathetic and parasympathetic neural control of heart rate is deranged in COPD patients and NIV acutely increases sympathetic response and decreases vagal tonus: the increase in ventilation induced by NIV is associated with reduced cardiac vagal activity in stable moderate-to-severe COPD patients [15]. As a matter of fact, it has been claimed that NIV may be useful to improve exercise tolerance also in CHF [16].

Table 1 shows the suggested pathophysiological mechanisms of NIV effect during exercise.

**Table 1:** **Suggested pathophysiological mechanisms for the effect of NIV during exercise**

**Setting**	**Mechanism**
**IPAP**	WOB reduction
	Respiratory Muscle unloading
	Limb blood flow switch
	Anti inflammatory action
	Reduction in Vagal Tone
**PEEP**	Reduction in iPEEP

Abbreviations: IPAP, Inspiratory Positive Airway Pressure; PEEP, Positive End Expiratory Pressure; iPEEP, Intrinsic Positive End Expiratory Pressure; WOB: Work of Breathing.

### Physiological studies

Different modalities of mechanical ventilation have been delivered non-invasively by nasal, facial Masks or mouthpiece during exercise [17].

#### Continuous positive airway pressure

Theoretically, CPAP should reduce the inspiratory threshold load on the inspiratory muscles of hyperinflated COPD patients and optimise neuromuscular coupling, thus improving dyspnoea and exercise tolerance [18-20]. CPAP counterbalances, at least in part, PEEPi (ie, the inspiratory threshold load) [21,22], reduces the WOB and increases exercise tolerance also in people with cystic fibrosis [23].

It has been suggested that the level of CPAP should be titrated on an individual basis to optimise benefit and comfort. Nevertheless, this implies the measurement of PEEPi, which is difficult if not impossible to perform on a routine basis [21]. CPAP seems also to be a useful strategy to improve functional capacity in patients with CHF, without any significant change in heart rate variability during physical exercises [24,25].

#### Inspiratory pressure support

Inspiratory Pressure Support (IPS) is a pressure-targeted mode in which each breath is triggered and supported by the patient [26]. This modality of mechanical ventilation is able to assist ventilation effectively when applied non-invasively in acute and chronic respiratory failure. Pressure support can improve dyspnoea and exercise capacity by reducing the high WOB of exercising COPD patients [27-33]. Patients with severe COPD can sustain exercise-induced lactatemia longer if assisted by means of IPS [34]. Using near-infrared spectroscopy and impedance cardiography to assess changes in cerebral oxygenation and cardiac output, it has been found that non invasive IPS plus positive end expiratory pressure (PEEP) added benefit to hyperoxia during exercise in improving central haemodynamics and cerebral oxygenation in COPD patients with exercise induced desaturations [35]. It has been also reported that during the walking test on treadmill of cystic fibrosis patients, non invasive IPS may change thoraco-abdominal kinematics and lung function, improving exercise capacity suggesting that NIV may be an effective tool to increase functional capacity also in children and adolescents with cystic fibrosis [36]. Non invasive IPS has been used also to improve exercise tolerance in patients with restrictive ventilatory pattern [37,38].

#### Proportional assist ventilation

Proportional Assist Ventilation (PAV) is a mode of partial ventilatory assistance endowed with characteristics of proportionality and adaptability to the intensity and timing of spontaneous ventilatory pattern by providing inspiratory flow and pressure in proportion to patient’s effort [39]. PAV was proposed as a modality of mechanical ventilation to improve the patient-ventilator interaction by bringing one of the two oscillatory pumps, the mechanical ventilator, under the control of the other, the patient’s central control of breathing [39]. Despite the positive characteristics, this modality of mechanical ventilation is still not used routinely in ICU. Several laboratory studies show that also non invasive PAV can increase exercise capacity of COPD patients without any relevant haemodynamic effect [40-44] in idiopathic pulmonary fibrosis (IPF) and obesity [45,46].

In a comparison study [45] submaximal test was increased in IPF patients with PAV compared with CPAP and without any ventilatory support. IPF patients performing submaximal exercise with PAV presented a lower heart rate during exercise, although systolic and diastolic pressures were not different among submaximal tests. PAV applied during exercise led to increase in exercise endurance and reduction in dyspnoea also in obese patients [46]. In these patients there was a large spectrum of response to PAV, with more than 50% of patients increasing their exercise endurance by a mean of 31%, thus suggesting that PAV might be a novel treatment option to enhance exercise capacity in a subgroup of obese patients in rehabilitation programs.

#### Controlled mechanical ventilation

Assist-control ventilation is a combination mode of ventilation in which the ventilator delivers a positive pressure breath at a preset tidal volume in response to a patient’s inspiratory effort. The ventilator also delivers breaths at a preset rate if no patient effort occurs within the preselected time period [47]. This modality of assisted ventilation was delivered through nasal masks during exercise in patients with pulmonary tuberculosis sequelae and kyphoscoliosis, resulting in a significant improvement in breathlessness and increase in exercise endurance [48,49]. Its use was not studied in COPD patients.

### Non invasive ventilation in pulmonary rehabilitation programs

The message of these physiologic studies can be summarised as follows: acute NIV, either as CPAP, IPS, or PAV, during exercise reduces WOB and dyspnoea increasing exercise tolerance in COPD patients and with less level of evidence in restrictive disease patients [6]. The next step is to evaluate whether NIV can be used as an aid during exercise training sessions. This is an important issue, because most patients with moderate to severe COPD are candidates for pulmonary rehabilitation programs (PRP) [4], and PRP are component of the comprehensive management of majority of patients facing lung transplantation or lung volume reduction surgery [50,51].

One randomised controlled study [52] found no additional benefit from providing NIV (in PAV modality) during exercise training on bicycle, on exercise tolerance, dyspnoea, or health status when compared with training alone. At difference, another similar study [53] found that after a period of PAV-assisted training, mean training intensity, and peak work rate were higher than after unassisted training. Iso-workload lactataemia after training was reduced to a greater extent in the assisted than in unassisted patients. Moreover, a significant inverse relationship was found between reduction in iso-workload lactatemia after training during the constant work rate test and peak work rate achieved during the last week of training. This was considered as a marker of true physiologic training effect.

In another study COPD patients were randomised into three groups for an intervention of 12 weeks: exercise training alone, NIV alone and combined treatment. All exercise capacity parameters improved after training and the combined treatment. In addition, peripheral muscle strength and six-minute walk distance (6MWD) increased after NIV. Levels of interleukin 8 and tumour necrosis factor α decreased after ventilation, and interleukin 8, C-reactive protein and surfactant protein D decreased after training, while all four of these inflammatory markers fell after the combined treatment [54].

Other investigators have confirmed benefits of performing NIV during exercise training as a part of PRP [55-57].

There are some potential problems with the use of NIV in PRP:

1. *Interface*. During high-intensity exercise, some patients may breathe through their mouth rather than (or together with) their nose, therefore they may require a full-face mask or a mouthpiece. Full-face masks may be more uncomfortable than nasal masks, potentially reducing the patient’s compliance to the procedure.
2. *Comorbidities*. Most of COPD patients have significant comorbidities, including a high prevalence of ischaemic cardiac disease [58]. The relief of exercise dyspnoea induced by with this kind of “mechanical doping” [59] might allow patients to exercise at a higher intensity and, as such, expose unaware ischaemic COPD patients to a load greater than their coronary ischemic threshold.
3. *Practicalities*. In the study [52], there was a high rate of drop-outs resulting from lack of compliance in the group under NIV. Furthermore, a mean 17-minute time spent to set the ventilator and supervise the training sessions with NIV was another practical drawback to the use of NIV during a high-intensity training program. It might not be practical or worthwhile to submit patients to unpleasant equipment (e.g., mask and related troubles), to the need for constant supervision of an individual operator to check for leaks and to reset the ventilator when needed and to a substantial risk for lack of patient compliance. The one-to-one patient therapist ratio increases the costs of the PRP. In these days of resource constraints this is a particularly important concern, especially comparing with the recognised benefits of well-conducted standard exercise training programs not requiring complex and sophisticated machinery or a personalised physiotherapist [4,6,59].

### Night time NIV and day time exercise

Severe stable COPD patients undergoing home nocturnal NIV and daytime exercise training showed a significant improvement in exercise capacity and health related quality of life compared with patients undergoing exercise training alone [60-62].

Furthermore, it has been reported that, in chronic hypercapnic COPD under long-term ventilatory support, NIV can also be administered during walking, resulting in improved oxygenation, decreased dyspnoea and increased walking distance. Therefore, NIV during walking could prevent hypoxia-induced complications [63,64].

Non invasive ventilation improves exercise tolerance in patients with acute exacerbations of chronic respiratory diseases, but the applicability of this approach in routine clinical practice may be limited [65,66]. In patients with restrictive disorders exercise training including NIV was shown to be feasible at home, whatever the modalities [67]. NIV alone is better than supplemental oxygen alone in promoting beneficial physiologic adaptations to physical exercise in patients with severe COPD [68]. Recently, wearable devices have been introduced to improve exercise tolerance of COPD patients [69].

## Conclusions

Two recent meta-analyses concluded that, given the small number of available studies, the small sample sizes, and the complete absence of power calculation, there is the need of randomised clinical trials with larger sample sizes based on statistical power calculations and designed to investigate the effect of training duration and intensity on rehabilitation [70,71].

As application of NIV in rehabilitation programs is a demanding tool in terms of heath resources, there is the need to identify the best indications. Several questions remain open:

1. Who is the ideal candidate ? Differences in results of different studies may be explained on the basis of different case mix, and apparently no study has directly addressed the topic of the best indication. Further studies are needed to identify the pathology (obstructive vs restrictive; hypoxaemic vs normoxaemic; hypercapnic vs normocapnic), the patient type, (naive to NIV vs acclimated to NIV; more vs less severely compromised patients).
2. Which are the most effective protocols? (e.g. training alone, vs nocturnal NIV, vs NIV under exercise).
3. Which are the most appropriate outcome indices to evaluate results? (e.g. tolerance to settings and equipments; incremental vs endurance test vs field test; Health Related Quality of Life vs dyspnoea vs daily life activities).

Despite promising results, for the above reasons and until the above questions are not answered, the authors of this review believe that the use of NIV during exercise training programs is unlikely to have a role in routine pulmonary rehabilitation settings. The use of NIV during exercise training as a component of PRP should be reserved to individual cases.
